# Integrating innovations: a qualitative analysis of referral non-completion among rapid diagnostic test-positive patients in Uganda’s human African trypanosomiasis elimination programme

**DOI:** 10.1186/s40249-018-0472-x

**Published:** 2018-08-18

**Authors:** Shona J. Lee, Jennifer J. Palmer

**Affiliations:** 1 Centre of African Studies, University of Edinburgh, George Square, Edinburgh, EH8 9LD UK; 2 Health in Humanitarian Crises Centre, London School of Hygiene & Tropical Medicine, Keppel Street, London, WC1E 7HT UK

**Keywords:** Human African trypanosomiasis, Sleeping sickness, Uganda, Passive screening, Diagnostics, Case detection, Referral completion, Rapid diagnostic tests, Elimination

## Abstract

**Background:**

The recent development of rapid diagnostic tests (RDTs) for human African trypanosomiasis (HAT) enables elimination programmes to decentralise serological screening services to frontline health facilities. However, patients must still undertake multiple onwards referral steps to either be confirmed or discounted as cases. Accurate surveillance thus relies not only on the performance of diagnostic technologies but also on referral support structures and patient decisions. This study explored why some RDT-positive suspects failed to complete the diagnostic referral process in West Nile, Uganda.

**Methods:**

Between August 2013 and June 2015, 85% (295/346) people who screened RDT-positive were examined by microscopy at least once; 10 cases were detected. We interviewed 20 RDT-positive suspects who had not completed referral (16 who had not presented for their first microscopy examination, and 4 who had not returned for a second to dismiss them as cases after receiving discordant [RDT-positive, but microscopy-negative results]). Interviews were analysed thematically to examine experiences of each step of the referral process.

**Results:**

Poor provider communication about HAT RDT results helped explain non-completion of referrals in our sample. Most patients were unaware they were tested for HAT until receiving results, and some did not know they had screened positive. While HAT testing and treatment is free, anticipated costs for transportation and ancillary health services fees deterred many. Most expected a positive RDT result would lead to HAT treatment. RDT results that failed to provide a definitive diagnosis without further testing led some to question the expertise of health workers. For the four individuals who missed their second examination, complying with repeat referral requests was less attractive when no alternative diagnostic advice or treatment was given.

**Conclusions:**

An RDT-based surveillance strategy that relies on referral through all levels of the health system is inevitably subject to its limitations. In Uganda, a key structural weakness was poor provider communication about the possibility of discordant HAT test results, which is the most common outcome for serological RDT suspects in a HAT elimination programme. Patient misunderstanding of referral rationale risks harming trust in the whole system and should be addressed in elimination programmes.

**Electronic supplementary material:**

The online version of this article (10.1186/s40249-018-0472-x) contains supplementary material, which is available to authorized users.

## Multilingual abstracts

Please see Additional file 1 for translations of the abstract into the six official working languages of the United Nations.

## Background

Human African trypanosomiasis (HAT, also known as sleeping sickness) is a fatal but treatable disease transmitted by tsetse flies. Owing to the weakness of health systems in areas where HAT occurs, an unspecified number of HAT cases still go undetected and unreported [1]. A key reason why HAT is under-detected relates to the complexity of diagnosis and treatment. The symptoms of HAT are typically intermittent, progressive and can be confused with other locally endemic diseases such as malaria, tuberculosis, or HIV infection which may also coexist with HAT [2]. Although trial results for new oral drugs in development suggest treatment regimens may become simpler and safer in future [3, 4], the need to manage adverse reactions and the costs associated with providing treatment^1^ means that patients suspected to have HAT based on symptoms are not treated presumptively. Microscopy-based examinations which confirm infection by allowing the parasite to be visualized in body fluid are therefore necessary, but are laborious and not very sensitive. Screening tests that identify trypanosome-specific antibodies or parasite DNA or RNA suggestive of infection may therefore also be used to complement microscopy in a variety of sequences, depending on disease prevalence and a control programme’s access to laboratory resources [6].

Mobile teams, which have been extensively used to screen at-risk populations in epidemics throughout the twentieth century [7], typically travelled with all laboratory equipment needed to confirm a case who would then be treated in hospital. In non-epidemic scenarios when mobile teams are regarded as too expensive and struggle to achieve sufficient coverage [8, 9], programmes typically revert to a passive case detection strategy with diagnosis restricted to places, usually hospitals, which can similarly perform all screening and confirmation tests in sequence [9–12]. In the rural areas where HAT is most endemic, however, such well-equipped hospitals are rare.

To improve the passive surveillance capabilities of countries seeking to eliminate HAT, programmes across Africa have recently devised novel case detection strategies to take advantage of new diagnostics which are easier to use and/or to build laboratory capacity to take advantage of tests previously considered too sophisticated for field settings [10]. Serological rapid diagnostic tests (RDTs) are an example of a diagnostic which is easy enough to be used in frontline primary healthcare facilities without electricity, cold chain or specific laboratory expertise. However, unlike malaria RDTs for example, HAT RDTs detect circulating antibodies, and therefore remain “screening”, rather than “diagnostic” tests in the strict sense. Their imperfect specificity also means that at typical elimination prevalences seen today they have a very low positive predictive value (PPV) [13] such that 99 false positives are produced for every true case [14]. Loop-mediated isothermal amplification (LAMP) which identifies parasite DNA [15], as well as the trypanolysis test which uses cultures of live trypanosomes to identify strain-specific antibodies [16] are examples of tests that are only performed in certain laboratories in Africa and Europe [17]. These can be used alongside RDTs to generate further evidence to increase diagnostic suspicion and demand for confirmatory tests.

By involving more health workers in HAT diagnosis at multiple health systems levels, this maturation in passive detection approaches addresses critiques in the HAT literature which view a move away from active screening as leaving the door open to disease resurgence based on health system weaknesses. HAT screening campaigns have sometimes been considered “vertical interventions (…) deployed in the absence of local healthcare infrastructure” [18] with the risk that the “progressive dismantling” of highly specialised mobile teams who possess the most expertise in HAT diagnosis could therefore have “grave consequences at the individual and community level” [14]. With diagnostic technologies now spread across three (or more) levels of the health system [10], however, this means that programmes must find innovative ways to manage a diagnostic algorithm for passive surveillance which is now routinely split up over geographic spaces. Either patients and/or samples will have to travel between health facilities and programmes will have to monitor these movements.

Previous innovations have focused mainly on transporting samples collected in medical surveys for remote screening to reduce the time full mobile teams have to spend in individual villages. This includes preserving blood samples in a stabilization buffer or as dried blood spots on filter paper for screening with the card agglutination test for trypanosomiasis (CATT) or its microCATT and latex agglutination variants [19, 20]; the indirect immunofluorescent antibody test (IFAT) [21, 22] and the trypanolysis test [13, 23, 24]. Positive results typically triggered a follow-up visit by a small, specialist mobile team. Sample collection for remote screening has, however, rarely been used in a passive surveillance strategy despite long-term recognition of the potential value of equipping frontline facilities to collect samples “at any moment” [21]. Although syndromic referrals have always been made from concerned health workers at ill-equipped facilities [25], until a few years ago, there were also few, if any, examples of programmes which asked serological suspects detected in a medical survey or frontline facility to travel to a different health system level for further testing [26, 27].^2^ Today there is a relative explosion of interest in piloting and studying the effectiveness of these technologies in new sequences and strategies [28, 29].

People who screen positive in these new passive screening strategies are implicitly expected to undertake a significant role in confirming (or disproving) their own diagnosis. Moreover, there is substantial work involved for both programmes and patients to make sense of discordant results, since patients who screen positive with RDTs but negative in subsequent tests must be followed up so they can either be confirmed or discounted as a case. Even without the extra layers of referral introduced by passive RDT-based systems, most HAT programmes typically achieve low levels of follow-up of persistent serological suspects in whom parasites cannot be identified but immune responses continue to be detected [17] or of treated patients to verify cure [8, 30].

In a rural context of material poverty, as in most HAT-endemic areas, structural and financial barriers play significant roles in treatment seeking decisions at each step of the passive surveillance referral pathway, beginning at the community level [11]. Patient motivation to keep seeking treatment for undiagnosed symptoms or to complete referrals may be severely diminished by high transportation costs, direct health care costs of recurrent treatment-seeking, competing family and agricultural responsibilities or restrictive employment systems for taking leave [25]. Age, gender and ethnicity of the patient [25], patient perceptions of severity and treatability of symptoms [31], and knowledge of treatment requirements [32, 33] may also influence referral completion. Moreover, unattractive aspects of the culture of care at receiving facilities produced by long waiting times, dismissive or harsh treatment by health workers, language barriers and recurrent drug stock-outs can dissuade patients from completing referral [34, 35]. Even if patients manage to reach facilities they have been referred to, there can be problems with patient processing and lab service unavailability [25] compounded by poor communication about referral linkages and poorly integrated recording and monitoring systems [36] that prevent referral consultations or tests from being carried out.

At each of these health systems levels, relationships of trust and power between patients and health providers is a key dynamic to understand programme compliance [37], with trust built partially on what people see and hear about technology (including diagnostics [38]) and institutions [39]. Thus, referral completion involves not only technical and organisational considerations, but also expectations and emotions [34]. Many studies of the referral process conclude that low referral completion reflects more about the health system than the patient, since “every non-respected referral is an unsatisfied patient with an expressed need but with an inadequate response of the health service” [35, 40] and suggest referral non-completion be defined in broader terms than simply the deviant, ‘non-compliant’ behaviour of patients [41]. For a referral-based intervention to succeed, patients potentially need trust in both referring and receiving facilities as well as programme supervision structures which support patients to move between them.

While the risks that referral non-completion poses to elimination programmes are widely recognised [14, 42], in depth qualitative studies on HAT referral non-completion from the perspective of patients themselves are few. In the West Nile region of Uganda which has recently decentralised its passive surveillance system, we examined patient experiences and perceptions of HAT, HAT tests and each of these parts of the health system in relation to HAT testing, to identify systemic challenges to referral completion by HAT screening suspects.

## Methods

### Context

**Fig. 1:**
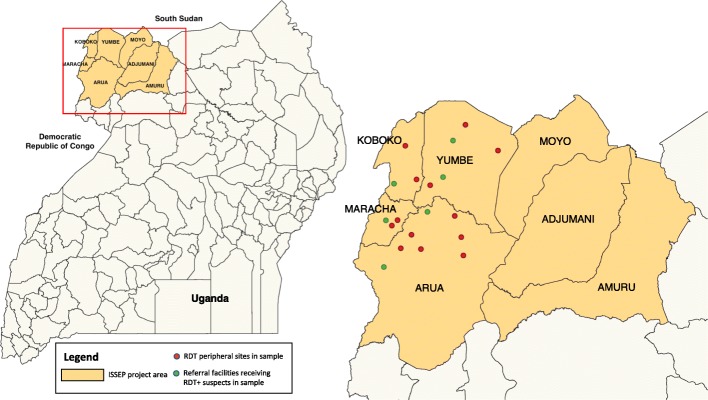
Map of West Nile region in Uganda showing locations of HAT-endemic districts included in the ISSEP and locations of referring (red) and receiving (green) health facilities included in the study sample. (SHP file obtained from public repository [58] and GPS coordinates of facilities included in the ISSEP taken from interactive online map of HAT diagnostic facilities [58])

Decades of wide-scale active and passive population screening using the CATT test with standard microscopy techniques as well as vector control reduced the number of reported *Trypanosoma brucei gambiense* HAT cases in Uganda from a peak of 1123 cases in 1997 to only 9 in 2013 [43]. Owing to the costliness of active screening in such a low prevalence context and the availability of new diagnostic technologies, the national control programme has since switched to an enhanced passive surveillance strategy [10]. Under the donor-funded Intensified Sleeping Sickness Elimination Programme (ISSEP, now called Trypa-No!) [29], between August 2013 and February 2014, the Ministry of Health introduced three new diagnostic technologies at increasing levels of the public health system across seven districts in the West Nile region of north west Uganda (see Fig. 1). All 212 health facilities in areas of West Nile believed to be at risk of *gambiense*-type HAT transmission were supplied with HAT RDTs [10].^3^ Nine well-maintained and staffed facilities in the project area were trained and equipped with fluorescent microscopes to enhance parasitological visualisation in blood samples. Three of these facilities were also upgraded to perform LAMP testing.^4^ The programme also conducted community sensitisation to circulate knowledge of the tests among the public during the first year of the programme, through meeting community leaders and radio broadcasts [10].

**Fig. 2:**
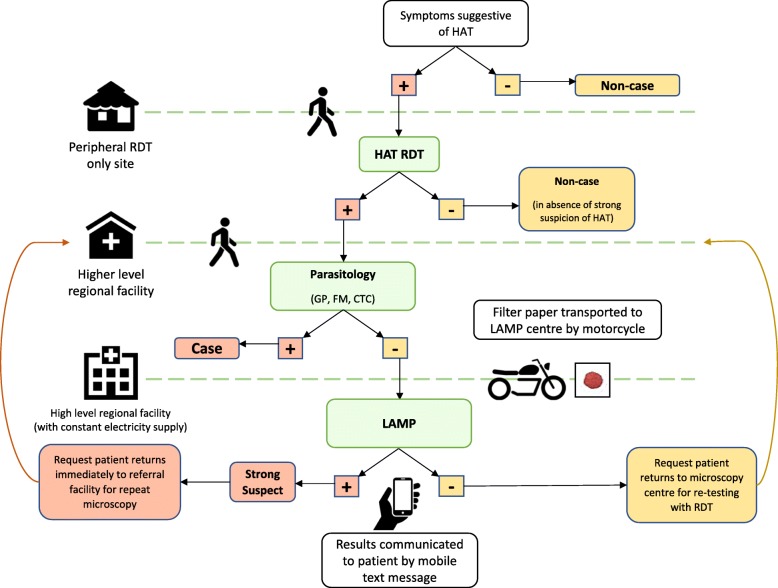
The *Trypanosoma brucei gambiense* human African trypanosomiasis diagnostic referral algorithm implemented by the ISSEP in Uganda. Higher level facilities have all diagnostic technology available at lower level facilities. Suspects must undertake key referral steps through the health system themselves. Adapted from Wamboga et al. 2017:7 [10]. Note: RDT: Rapid diagnostic tests; HAT: Human African trypanosomiasis; GP: Glandular puncture; FM: Fluorescence microscopy; CTC: Capillary tube centrifugation; LAMP: Loop-mediated isothermal amplification

Health workers were trained in syndromic suspicion of *gambiense* HAT, and on the new diagnostic algorithm (see Fig. 2). In it, only patients with symptoms suggestive of HAT are offered the RDT. Patients who exhibit symptoms also suggestive of malaria are first tested with a malaria RDT. If a negative result for malaria is recorded, or a positive result but symptoms persist after one week of treatment, then a HAT RDT is used. Patients who screen positive with the HAT RDT (‘RDT+ suspects’) are referred to the closest facility where parasitological investigations can be carried out such as cervical gland puncture (GP), blood smear staining for use with fluorescent microscopy (FM) or capillary tube centrifugation (CTC) of blood to concentrate parasites in the buffy coat. If parasites are identified, patients are treated. Otherwise, a blood spot dried on filter paper is transferred by motorcycle to a facility where it can be tested using LAMP. As LAMP is currently an experimental test, hospitals are required to gain patients’ informed consent to this. LAMP results are communicated via mobile text message to the patient. If the LAMP test is positive, then the suspicion of HAT is strengthened and the patient must return immediately to have microscopy repeated. If both parasitology and LAMP tests are negative, then the patient is requested to return for a quarterly follow-up visit. Patients are then tested every three months with HAT RDTs until they become seronegative, or are confirmed as cases [10].

Although this programme reported high referral completion in its first year (see next section), the issue of suspects ‘defaulting’ on their referrals was described by programme staff as a significant challenge to their work. To address this while avoiding introducing unsustainable administrative and financial practices, the ISSEP granted programme staff at the district level substantial discretion about how to integrate monitoring and support of RDT+ suspect referrals into existing systems and activities. Most frequently this included direct telephone communication with patients, or communicating through local village health team members operating near the patient’s home to counsel patients to present for further testing. When repeated attempts to convince suspects to attend referral appointments failed, district supervisors themselves would arrange to travel to the patient’s home and collect them in person. In one district with a LAMP centre and high levels of referral non-completion, lab personnel were permitted to collect dried blood samples on filter paper for LAMP testing, inverting the formal diagnostic algorithm under a pilot approach.

### Patient sample selection

Quantitative patterns across the programme were established in July 2015 during a scoping investigation to target further enquiries; detailed quantitative and qualitative data on referral completion was then collected from four districts over a three-week period in November 2015. In this stage, supervisors provided lists of RDT+ suspects who had not completed referral (i.e., were considered ‘referral outstanding’), helped to select a purposive sample, and assisted in contacting the individuals in our sample.

By the end of June 2015 (20 months into the programme), 12 495 RDTs had been performed across the West Nile region, yielding 346 RDT+ serological suspects (2.77% seropositivity prevalence, Table 1).

**Table 1:** Cumulative (to end-June 2015) RDTs performed, suspects identified and suspects outstanding for referral, by district

District	RDTs performed	RDT+ suspects identified (all facilities)	RDT+ suspects (peripheral sites only)	RDT+ suspects outstanding for microscopy
Arua	3925	115	90	23
Maracha	1358	61	57	7
Koboko	2574	66	64	14
Yumbe	1847	39	24	3
Moyo	1921	33	17	1
Adjumani	368	19	5	0
Amuru	502	13	11	3
Total	12 495	346	268	51

*RDTs* Rapid diagnostic tests, *RDT+* RDT positive

Of these, 295 (85.3%) had completed at least one confirmatory microscopy visit. Among suspects identified only at peripheral RDT sites (excluding microscopy and LAMP centres where confirmatory testing of seropositive suspects can typically be done on the same day), this proportion was slightly lower at 81.0% (217/268). Ten HAT cases had been detected from all sites.

**Fig. 3:**
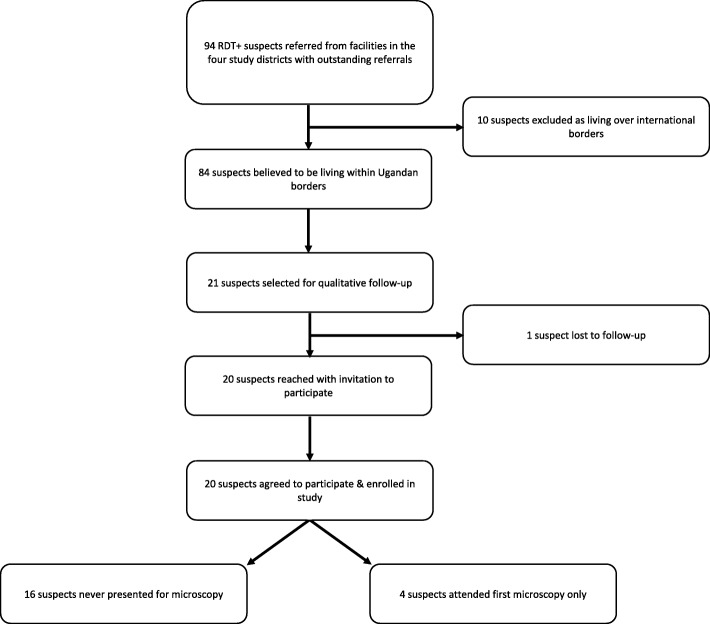
Sample selection process followed for qualitative interviews

Facilities in the eastern districts of Moyo, Adjumani and Amuru had reported low numbers of RDT+ suspects and correspondingly low numbers of outstanding referrals (*n* = 4 for the first referral step from all three districts in July 2015). Detailed investigations in November therefore focused on patients referred from facilities in the four western and central districts in the ISSEP with high numbers of RDT+ suspects outstanding for microscopy investigations: Arua, Maracha, Koboko and Yumbe (see Table 1, Fig. 1). Supervisors from these four districts were asked to draw-up a list of RDT+ serological suspects outstanding for any microscopy visit (*n* = 94 from beginning of programme to end-October 2015, see Table 2, Fig. 3).

**Table 2:** Demographic profile of all outstanding RDT+ suspects in four districts and those interviewed

	RDT+ suspects outstanding	
	Total identified, *n* (%)	Interviewed, *n* (%)
Total	94	20
District		
Arua	30 (31.2)	9 (45.0)
Koboko	22 (23.4)	2 (10.0)
Maracha	20 (21.3)	3 (15.0)
Yumbe	22 (23.4)	6 (30.0)
Gender		
Male	34 (36.2)	5 (25.0)
Female	60 (63.8)	15 (75.0)
Age (years)		
Median (range)	30 (3–79)	40 (8–76)
Time referral outstanding (months)		
Median (range)	12.9 (1.2–26.2)	13.6 (3.0–26.3)
Distance to referral facility (km)		
Median (range)	13.0 (1–50)*	15.0 (5–48)

*n = 92 as data on the referring facility was missing for 2 patients

Patients who had never reported for any microscopy visit were considered alongside those who had previously reported for microscopy but had not completed quarterly follow-up(s), as monitoring instruments had not been standardised by this time in the ISSEP and some district supervisors could not differentiate between these patient groups. Any patient reported by supervisors from previous follow-up attempts to be living over international borders (Democratic Republic of Congo or South Sudan, *n* = 10) was excluded from follow-up by the research team. From this reduced list of 84 suspects who had not reported for microscopy testing, 21 people were purposively selected for recruitment, aiming for a diverse mix of people chosen according to referring district and across categories of three key characteristics we hypothesised might influence referral completion: length of time since first screening RDT+, distance between the patient’s village and the microscopy centre they had been referred to, and subjective characterisations by district supervisors of the level of difficulty they had previously had counselling or reaching patients to complete referrals. Out of five suspects characterised as ‘difficult to convince’, four were selected for inclusion in the study and three could be located. Therefore, of 21 patients selected for the sample, 20 could be located; all of these consented to participate and were recruited (see Table 2, Fig. 3).

**Fig. 4:**
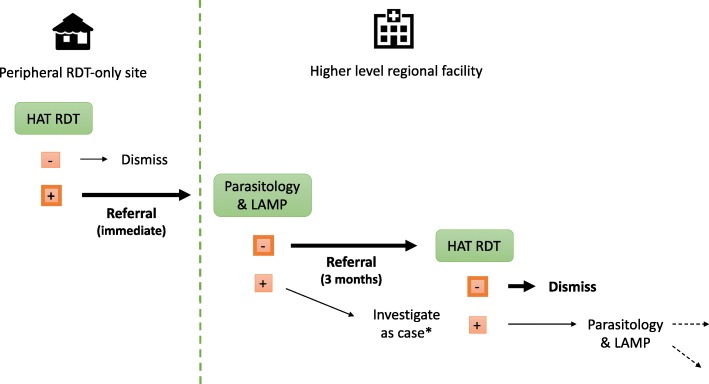
Diagnostic trajectory of patients in our study sample (indicated in bold) and most RDT-positive suspects in an elimination programme. The 16 patients we interviewed who had not presented for parasitology and LAMP testing within 1 month of referral ultimately (after the interview) tested negative on all subsequent tests. The 4 patients we interviewed who had presented for parasitology and LAMP testing but not for their quarterly follow-up RDT test ultimately tested negative and were dismissed. *Patients who test positive via parasitological tests are considered cases, while those who test negative but LAMP-positive are sent back for further parasitological testing

Sixteen out of 20 RDT+ suspects in our sample had not completed their first referral visit for microscopy/LAMP testing (at least one month had elapsed since screening RDT+, Fig. 4). Four had previously completed one microscopy visit but were outstanding for their follow-up RDT test (at least three months had elapsed since confirmatory testing); in these instances, analysis focused primarily on reasons for non-completion of the follow-up examinations, and this difference is indicated in the text as ‘RDT + MS- suspect’ for ‘microscopy negative’. All the suspects completed the necessary outstanding tests after interviews; no parasites were identified and all were dismissed from further evaluation.

### Recruitment

Suspects were mobilised for interviews two days prior by telephone and/or through village health team and local council representatives who explained the study. It was clarified that interviews would be conducted with a researcher independent of the programme and that transportation to a microscopy centre would be available on the interview day if patients wished to complete referrals. A letter of introduction containing information on the HAT testing referral process, the study, and telephone numbers for the mobiliser and research team was left with patients. Interviews took place at the microscopy centre while patients waited for confirmatory test results. For suspects under 18 years of age, adult guardians were interviewed in the presence of patients. No patients presented with psychiatric symptoms or mental changes that suggested they could not participate in interviews, per supervisors’ assessments. Verbal informed consent was obtained from patients on initial contact and written (or witnessed oral) consent was recorded before the interview.

### Interviews

Interviews followed a semi-structured interview guide which collected information on: suspects’ symptomatic course of illness and treatment-seeking; knowledge and awareness of HAT; awareness, understanding and opinions of HAT RDTs; understandings of RDT results; and experience of referral at all stages. This guide was pilot tested in interviews with people who screened positive during a concurrent mobile team-led screening campaign and who were later assembled at microscopy centres for confirmatory testing. It was also further refined during the study as new themes emerged. Interviews took place in English or through consecutive translation to local languages by trained interpreters, as necessary. Discussions were audio-recorded and full transcripts produced shortly afterwards, annotated with notes taken during and following the interview.

### Analysis

Descriptive statistics were produced in an Excel 2016 (Excel for Mac, version 16.10, Microsoft, US) spreadsheet to compare characteristics of respondents to the wider sample of people who had not completed referrals; distances between screening and microscopy sites were calculated using facility Global Positioning System (GPS) information available on the programme website [44]. Responses to each question from the interview topic guide were consolidated under headings in one document for primary analysis. Each participant was attributed a unique identification code with accompanying demographic characteristics to aid interpretation. Recurrent themes for each topic were then identified, and key quotes that articulated these themes were selected to summarise each before secondary analysis across themes. For each theme, we analysed responses from patients in the two referral completion groups (RDT+ versus RDT + MS-) separately, but combined them in the final analysis (except where indicated) when they did not substantially differ. Patients’ age and gender were removed before presentation to preserve anonymity. Quotations presented from study participants were occasionally edited to correct grammar for readability, while preserving the meaning and tone of the comments.

## Results

### Profile of RDT+ suspects interviewed

Of the 20 RDT+ suspects interviewed, more (15/20) were female, as in the wider 94-person sample of all outstanding suspects in the four districts (63.8%, see Table 1). The median age of people interviewed was 40 years old (range 8–76), higher than the median age (30 years old) of all outstanding suspects. The median time between first screening RDT+ and our interview was 13.6 months (range 3.0–26.3, 13.5 for the 16 RDT+ suspects and 16.6 for the four RDT + MS- suspects), similar to the median outstanding referral time of all suspects (12.9 months). Participants had been screened at 13 frontline facilities across the four districts (Fig. 1). The median distance from respondents’ RDT screening sites to the facility they were referred to was 15.0 km (range 5–48 km), slightly further compared to the whole sample (13.0 km, range 1–50).

### Circumstances leading to RDT testing

In all cases in our sample, the decision to use HAT RDTs was initiated by health workers, rather than on the request of patients. At the time of interview, most suspects reported having experienced symptoms consistent with HAT, particularly headaches, fever, or excessive sleeping during the day. HAT-like symptoms were commonly described as part of a long-term, difficult to diagnose or treat illness that some patients reported suffering from for years.

While peoples’ symptoms matched the HAT syndromic screening profile however, only one person, who had a family member previously treated for HAT, had ever considered they might have had the disease before being tested. Everyone else assumed that they were suffering from malaria or typhoid or were unsure of what could be causing their symptoms, so sought diagnosis and treatment from local health facilities and drug shops. Some additionally considered whether they might be affected by witchcraft or a common flu, and so took herbal treatment. The following illness history was illustrative:

*It started like malaria. From there I took a step and went to the clinic. I bought a drug, tablets. I took the drugs for two to three days, on the third day this thing threw me down, I was bed ridden […] from there they [health staff at an ISSEP facility] told me this is not malaria, what is detected it looks like sleeping sickness. (RDT+ suspect 19, Maracha)*

None of the 20 suspects interviewed reported to have requested to be tested for HAT themselves, as described by this patient: “The technician health worker started just removing blood and testing, and told me they have found sleeping sickness in my blood. It wasn’t my previous idea that I am coming to test for sleeping sickness” (RDT+ suspect 17, Koboko).

Partly, this may relate to the fact that before testing, awareness of the HAT RDT among respondents was very low. Only two people (both from Maracha District) had previous knowledge that the RDT was available at their local health centre through sensitisation activities. One respondent in Koboko District knew they were available in the main referral hospital.

### Awareness and feelings about HAT

While patients may not have suspected HAT in themselves before being tested for it, the majority of patients we spoke to appeared to take the disease seriously. This included an awareness of their own risk from HAT, particularly after receiving a positive RDT result.

Almost everyone interviewed had personal knowledge of HAT, having known relatives or people in their village who had suffered or died from it during outbreaks in previous years. Other key sources of knowledge included community sensitisation campaigns associated with medical active screening programmes or tsetse fly control interventions using insecticide treated targets.

At the time of interview, nearly all respondents claimed to feel there was a risk of HAT in their area. Peoples’ perceptions of risk were discussed in terms of their proximity to tsetse flies near rivers, ‘the bush’, and dark, densely vegetated forest areas. Risk was also interpreted in relation to the presence of HAT interventions, with comments such as, “We have that fear because the screening teams came to our village” (RDT + MS- suspect 1, Arua) and “I have fear in my hut, I have seen tsetse fly nets being hung up [nearby]” (RDT+ suspect 19, Maracha).

Given peoples’ lack of awareness of HAT RDTs, their presence in frontline facilities did not seem to have had a similar influence on suspects’ perception of HAT risk prior to testing. Comments about not personally feeling to be at risk *until* testing RDT+ such as the following, however, suggest that the process of screening RDT+ may have heightened some suspects’ perception of personal susceptibility to HAT: “from the result of my blood I have that thought that I have sleeping sickness” (RDT+ Suspect 21, Maracha).

### Experiences at referring facilities

At referring facilities, patients appeared to have received little information about the HAT testing process. In all cases the health worker they visited had tested them for HAT alongside tests for malaria or typhoid and most patients told us they only became aware that they had been tested for HAT after they received a positive RDT result. Five people (a quarter in our sample) even left the facility not understanding they had screened positive for HAT. Three of these people reported that the first time they heard they could be infected with HAT was when a district supervisor, lab supervisor or village health team member followed-up their outstanding referral, as described by the patients quoted below.

*That time he [the health worker] did not tell me he has found sleeping sickness in my blood, but he told me he has found malaria […] When these technicians from Yumbe hospital [a microscopy site] went to Kochi [an RDT site] they gave information to me at home that they have discovered sleeping sickness in my blood. I said, ‘why so abrupt like this?’ When I went for the test they didn’t tell me I had sleeping sickness. Even my husband had to pick my small patient book and went to hospital to check, and found that in my small book it was not indicated that I had sleeping sickness, but the big book had my name in the list with the names of people who have sleeping sickness, that is how I got to know about it. (RDT+ suspect 14, Yumbe)*

*I was not told I have sleeping sickness, no one in the health facility told me that until they wrote information and sent it through some guy who is doing business in this trading centre […]. He said, ‘did they tell you about it when you went for the test?’ I told him ‘no they did not tell me’. Now they have told me that I have sleeping sickness. (RDT+ suspect 5, Arua)*

Two suspects reported that sensitisation for our interview was the first notification they had received. Such patients therefore reported not to have known about any follow-up appointments, nor that they were considered to have an outstanding referral by the national programme.

Even when results had been given at the time of consultation, however, several people interviewed expressed confusion and even suspicion about why they were being referred. Only a minority of patients attributed the reason for referral to limitations of the test, as in the following excerpt:

“*They told me this could possibly be sleeping sickness. Since their machine’s detective strength is not adequate I should come to Omugo [a microscopy site]”. (RDT+MS- suspect 3, Arua).*

More often, rather than questioning the reliability of the RDT, peoples’ confusion about referral rationale was expressed as distrust in the expertise of referring health workers who appeared to not be interpreting the results correctly:

*I did not trust them because they told me that I should come for further testing in Omugo, which means they failed to interpret the result from the first test. (RDT+MS- suspect 4, Arua)*

*It was explained, but he did not explain in a direct way. He did it in an indirect way, saying that the drug for sleeping sickness is at Omugo or Arua [another microscopy site], so you must go there to get the treatment. (RDT+ suspect 4, Arua)*

*People who are learned, they automatically use politics in their speaking. He [health worker] did not tell me exactly the way you have said, but he showed me the way so that I can come and discover from this side (RDT+ suspect 17, Koboko).*

*When these people told me that I could be having sleeping sickness I felt they are not being open to me, I was trying to force them to be open. I thought that if they know that it is there, they should tell me I have sleeping sickness, so I was trying to force them. I knew I had sleeping sickness [because] I would be among other people and I would fall asleep during the day. That is the symptom I knew meant I had it. (RDT+MS- suspect 2, Arua)*

As evident from the last quote, patients’ own interpretations of their symptoms influenced their trust of test results and health worker interpretations of them.

While some were sceptical that they could have HAT because their illness experience was inconsistent with what they had heard about the disease, others trusted RDT results because they were “feeling it inside [their] blood” (RDT+ suspect 10, Yumbe) or in some other way, saying, for example: “My swollen legs did not change, and the signs and symptoms I experienced continued, so I believed I had sleeping sickness” (RDT+ suspect 2, Arua).

### Expectations of receiving facilities

Before receiving their results at microscopy centres, patients reported that they would likely trust the second round of tests more than the first, associating more reliable tests with being conducted in larger hospitals and requiring larger amounts of blood:

*Because I think this is the biggest hospital that can bring out the truer result than the previous one (RDT+ suspect 12, Yumbe)*

*It can differentiate between truth or lies. So if the first test may say it is true I have sleeping sickness, while today it might say it is false, or the first test might say I don’t have, while here it will say that I have; I will prove from here (RDT+ suspect 17, Koboko).*

Only one patient expressed distrust of the motivations behind systems in larger hospitals. One RDT + MS- patient told us she had declined to come for further blood tests because of family members’ suspicions about blood stealing, saying:

*“I got false information from people that they had come to steal my blood, so I was not in a position to come. I spoke to my people at home, but my husband was not pleased so I would not have come by myself” (RDT+MS- suspect 2, Arua).*

Other characteristics associated with receiving facilities, besides trust in the tests they offered, thus appeared to influence most peoples’ decisions not to present, particularly financial concerns related to referral.

RDT+ suspects frequently cited the cost of transportation and the difficulty of leaving children at home as a burden associated with travelling to microscopy centres. Many people therefore admitted they would likely not attend a further follow-up appointment if unaided by the programme. Patients also worried about expenses at the receiving facilities such as ancillary fees for tests and inpatient care if infection was confirmed.

Though HAT tests are provided free of charge at all levels of the health system, lab fee structures in Uganda have inconsistent policies across diseases, so patients referred for many tests will normally be charged for at least some of them and some facilities charge a general lab fee on top of fees for individual tests. This helps explain one man’s story about deciding not to complete his HAT referral on a previous trip to the receiving facility. He said:

*When I was taken to Arua, I was to be tested for sleeping sickness but these people charged me 20,000 shillings. Because I did not pay the 20,000 they did not test for sleeping sickness, I had to come back home. I had already got the result that I have sleeping sickness in Siripi [health centre, an RDT site] but I was referred for further tests in Arua. They charged 20,000 because they said they [in Siripi] could not do the second test from there (RDT+ suspect 4 Arua).*

Similarly, very few people were aware that HAT treatment was free of charge. Patients also anticipated the costs of food and some in-hospital care costs not covered by the sleeping sickness programme if admitted for treatment the same day, with one woman saying:

*I need to make sure there is money for transport and money for feeding. When you are found positive and you need to be admitted then you also need food and someone to stay around you. Since I’m just like this by myself and I have small children, they are not strong enough to look for things to help me so I could not come (RDT+ suspect 6, Arua).*

One suspect additionally worried about the indirect costs of treatment even after discharge from hospital, saying:

*“When you are vaccinated with that vaccine [referring to the lumbar puncture used for disease staging] you cannot work […] I also heard that this treatment would take long in your body and you would fail to recover, especially this treatment will fail to treat you because curses are attached to your life” (RDT+MS- suspect 3, Arua).*

### Dealing with discordant results

Receiving discordant (RDT + MS-) results at labs in receiving facilities which required further diagnostic referral steps caused some patients to revise their understanding of which tests could now be trusted to give the ‘true result’. As one patient described:

*I thought that Omugo [Hospital, a microscopy centre] has to be the one to give the true result […] but they told me it takes a long process to come up with a result, so I have to come back and they will send the result here and the technician will tell me if I am truly sick (RDT+MS- suspect 1, Arua, referring to the process of further testing via LAMP)*

For others, like at referring facilities, some peoples’ confusion was expressed as distrust in the health staff that performed their microscopy. This was especially true for the RDT + MS- suspects whose referrals were outstanding in our sample. One patient said: “since they did not cure me, I am not sure of their profession, I’m not sure of their work […] the health worker, the one who tested me did not discuss the result with me, instead they discussed it with another health worker” (RDT + MS- suspect 4, Yumbe).

Realising the financial implications of more travel associated with conflicting results gave patients the impression that completing all confirmatory testing would become a very expensive process. Many patients also highlighted the unfairness of their compliance with HAT programme referral rules but not, in turn, being taken of care of by the same system. One person, for example, demanded to know from us, “Now that you have brought us, after testing, will they give us treatment straight away or not?” (RDT + MS- suspect 2 Arua). Another suspect explained: “First I came there and was found positive, and from here I was told the disease is not there so was told to come after three months, so I was taken to the other unit and was on some medication. I took all those drugs, but *still there is no change*” (RDT + MS- suspect 1, Arua, italics authors’). Such suspects, who believed that they did indeed have HAT, strongly disliked that HAT treatment could not be given at the moment of testing, as for other diseases such as malaria. One suspect while awaiting their microscopy results explained, “because the symptoms I experience still continue, I expect that today I will be given some drugs to take home” (RDT+ suspect 2, Arua).

We observed very few (only four) instances of serological suspects receiving further clinical investigation for symptoms after microscopy or repeat RDT testing. Typically, when RDT+ patients arrived at microscopy centres, clinical staff were briefly called out of the outpatient department to obtain consent for sending a blood sample for LAMP testing in the likely event that they tested negative via microscopy (RDT+ patients) or to record that a suspect had returned for follow-up (RDT + MS- patients). While clinical staff appeared dedicated to the ethics consent counselling process, their time was limited and a full syndromic examination and exploration of alternative diagnoses did not seem to be part of their usual routine. Moreover, long outpatient queues of up to 1 h required to see clinicians after testing negative meant that many patients preferred to return home and (for RDT+ patients) wait for LAMP results by phone.

## Discussion

The development of RDTs to screen for HAT in recent years changes the possible configurations of passive surveillance and care in health systems. To understand how a change towards decentralised testing affects patients, we interviewed a sample of people who had visited a frontline facility in Uganda with symptoms suggestive of HAT, screened positive with RDTs there but failed to present for a further testing step at higher-level facilities. Everyone in our sample was eventually determined not to have the disease. While true cases with more severe symptoms might be expected to more closely comply with referral instructions, the majority of patients we interviewed appeared to take HAT seriously, including their own risk from it, particularly when receiving a positive result from a HAT RDT. They also largely trusted the effectiveness of tests positioned at high level facilities. So how should we understand their failure to comply with referral instructions?

A HAT surveillance system which relies on referral through all levels of the health system will inevitably be subject to its limitations. We identified issues at both referring and receiving facilities as well as issues with navigating between them which made completing referrals challenging for patients.

At referring facilities, there were clear problems related to communication about the process of testing. The majority in our sample were not aware they had been tested for HAT with RDTs until receiving the result. Power imbalances between health providers and patients have been suggested to contribute to a culture of poor communication around HIV testing in Uganda, whereby many patients are tested without their knowledge and miss opportunities to discuss testing rationale with health staff [45]. In our study, while everyone we interviewed had been actively seeking care for their symptoms when tested, most patients never considered HAT as a possible diagnosis for themselves and most were unaware of the possibility that they could be screened for HAT at their local health facility, so HAT-testing in our sample was entirely health worker-led. This may be a characteristic peculiar to our sample. In a study of 49 HAT referral decisions narrated by health staff in this programme, one-fifth were prompted by patients themselves [46]. Another study of treatment-seeking trajectories of 877 people screened passively for HAT (including 38 confirmed cases) in South Sudan identified lay person-initiated referral as the most common process associated with screening and successful detection [25]. If self-referral for RDT testing is a common behaviour in this programme, greater dedication to referral completion in this type of patient might contribute to the high overall completion rates in the programme.

Relatedly, since communication problems continued even after patients screened positive for HAT, it raises the question of whether communicating about referral is more complicated when testing is initiated by health workers. A quarter of suspects interviewed, for example, reported leaving the facility without knowing they could have HAT –either because referral messages were not given or not understood. Heavy staff workloads have been shown to hamper post-test counselling to HIV-positive patients [47] and contribute to pre-antiretroviral treatment drop out in Uganda [48]. Health workers in the ISSEP likely had difficulty communicating with patients about their HAT testing intentions quickly and easily because of similarly high workloads. Low awareness about HAT RDTs among the treatment-seeking population also presumably compounded this.

After leaving facilities, some people in our sample who had left not knowing they had HAT referrals to complete were reached by messages from supervisors or village health teams. While it was clear that some patients in our sample felt uncomfortable receiving healthcare directions outside of health facilities this way, it is also likely that without the flexible methods and personal motivation of supervisors to ensure programme objectives were met, the programme would otherwise not have seen such high referral completion rates. The ISSEP’s introduction in 2016 of an information system which automatically sends reminders to patient’s mobile phones to go for testing will presumably also address some of this communication gap [10].

Patients also highlighted the direct, indirect and opportunity costs associated with travel which provided practical challenges moving between facilities which are well-recognised in public health literature [25, 37]. With patients outstanding for referral living a similar median distance away from receiving facilities as all people in the programme, most of whom managed to complete them (13.0 km in the 94 person sample in our study and 15.0 km in the 20 people we interviewed versus 12.5 km in the programme overall [10]), however, transportation issues alone don’t explain non-compliance.

At receiving facilities, some patients anticipated fees associated with confirmatory testing, particularly if they were referred for more than just a HAT test. Additionally, many patients anticipated substantial indirect and opportunity costs associated with a hospital stay for HAT treatment. Patients talked about delaying referrals because they needed to raise money for the hospital stay over and above transport costs, suggesting either an abundance of caution in preparing for the chance that they would be identified as cases or that patients conflated a seropositive RDT result with being a case and needing treatment. The latter explanation is more problematic but has conceptual precedents.

Under the mobile team-led system of HAT screening which preceded the RDT’s introduction, all patients who screened seropositive via the CATT test immediately underwent confirmatory testing and any confirmed cases identified were offered transportation back to a hospital for disease staging and treatment. Patients who could not travel on the same day could report for staging and treatment in their own time. Compared to the RDT-based algorithm now in use, the CATT-based algorithm also produced far fewer serological suspects who required follow-up,^5^ so very few people were asked to travel unless they were confirmed HAT cases. But in an elimination phase, regardless of which screening tests are used, the proportion of false positives will continue to increase given the inherent cross reactivity of antigens used in these tests with other parasites.

Another precedent relates to peoples’ prior experience with malaria RDTs which prefigured the appropriateness of RDT technology as the basis for the new HAT case detection strategy in Uganda as staff were already familiar with malaria RDTs and implementing their referral algorithms [10]. In most settings where RDTs were introduced they replaced the need for microscopic examinations in malaria; today, a positive malaria RDT result normally immediately leads to treatment in the same facility as the test was done, while a negative result triggers alternative pathways of care toward further diagnostic procedures or referral to higher level facilities [38]. If patients are sent elsewhere for malaria treatment, this is typically only because the facility is out of stock of drugs –something that is known to harm peoples’ trust in public facilities for malaria [49]. Thus, it is reasonable to assume that few people familiar with either of these precedents would have expected to be sent home without receiving treatment after a HAT referral.

Cutting across the system, there were also critical issues around patients’ interpretations of the contingent nature of positive HAT RDT results which necessitate referral, and the related issue of discordant (RDT + MS-) results between tests. Importantly, discordance is the most common outcome for serological suspects who complete referral in a HAT elimination programme given the difference in performance between the different HAT tests currently being used. None of the suspects in our study were confirmed as cases after microscopy. Without support to interpret results, however, several patients felt the truth of their diagnosis was somehow being concealed from them or treatment was being denied because health staff were incompetent or behaving evasively. Patients’ own understanding of their symptoms also sometimes influenced this perception. There is a long history of patients avoiding programmatic directives in Uganda because they are suspicious of the motives of health staff [50, 51]. A conceptual discordance for patients thus emerged not only between their trust in different types of tests but also in their trust between tests and health workers –or more accurately, the legitimacy underlying each. Trust in health workers or institutions can also be undermined when falsely-positive suspects do not receive an alternative diagnosis and treatment for their symptoms when repeatedly presenting to microscopy centres at great cost to themselves. Studies of malaria RDTs have shown that health providers working in under-resourced laboratories are aware of this risk and compensate by returning falsely positive results (and unnecessary treatment) to satisfy patient expectations for an easy to manage diagnosis and to avoid accusations of incompetence [38].

Community perceptions of HAT control programmes are not only influenced by historical memories of past interventions, but shaped by how new methods are introduced. It is unrealistic to expect that community perceptions will unreservedly accommodate new interventions without thorough information dissemination which includes two-way communication between communities and programmes [52]. Studies of HIV diagnosis have shown that the likelihood of patients accepting testing increases with thorough explanation of the testing processes, testing venue and understanding what needs to be done after receiving test results [53]. While all technical aspects of HAT tests may not need to be communicated to patients, false positive or discordant results should be acknowledged as a normal and expected outcome so as not to harm health workers’ reputations. Health personnel must clearly explain to suspects what a positive result from a HAT RDT really means while stressing the need for confirmatory testing: that it is an indicator of possible exposure to HAT and that RDT positivity alone does not confirm one as a HAT case. Moreover, beyond advertising the availability of HAT RDTs, HAT programmes should clarify how the HAT referral system differs from diagnostic systems for other diseases to avoid unmet expectations stemming from similar language but different diagnostic meanings shared across the wider health ecosystem [54].

The splitting of passive surveillance diagnostic algorithms across multiple levels of the health system undoubtedly adds layers of potential complications to patient management. Not only are more cadres of health staff now involved in making sense of discordant results, technical differences between strategies relying on the RDT compared to the CATT may even create more discordant serological suspects for the system to deal with as discussed above. As more RDTs come on to the market, trials are underway to test strategies which use diagnostics in different combinations, including the use of different RDTs in tandem to improve case detection performance and the use of sample collection for remote screening at lower health system levels to reduce patient travel [28].

HAT elimination surveillance programmes are understandably interested in optimising sensitivity since any missed case can be a potential source of infection from which epidemics can start to build. On the other hand, this study has drawn attention to the human cost of imperfect test specificities in a context of low disease prevalence. Moreover, that so few patients in our sample left microscopy facilities with an alternative diagnosis or treatment for their ongoing symptoms raises an important discrepancy between meeting the objectives of an elimination programme and meeting individual patients’ needs. Failure to address the latter may have detrimental effects on referral adherence in the healthcare system as a whole.

Because of our study design we cannot say what drives referral completion success in this programme which achieves a remarkably high proportion of completed referrals (85%). By studying the experiences of referral completion failure however, we show how, in many ways, access to HAT testing remains fragmented and logistically challenging for patients, despite the higher coverage of screening tests across the system. Additionally, it is unlikely that the health systems issues we have identified solely affect people who did not complete referrals and may be especially important for HAT programmes operating in places with weaker health systems to understand. Completion proportions may furthermore diminish for patient groups at each stage of quarterly follow-up referral and should be monitored as programmes mature.

## Conclusions

While the literature on the role of diagnostics in HAT elimination largely focusses on coverage and identifying remaining cases in the population, our study brings to light the mundane work involved in managing the large numbers of non-cases produced by an imperfect surveillance system. Furthermore, rather than focussing only on patients’ health-seeking behaviours in the diagnostic process [55], we turn our attention to the practicalities of the entire diagnostic ecosystem [54] and the structural processes involved in reaching a confirmed HAT diagnosis. By focussing on how HAT RDTs fit into the wider health system, we have also shown some of the ways that introducing a new RDT can destabilise and disrupt an established diagnostic ecosystem by creating additional layers of bureaucracy, further tests, travel, and work for patients and health workers.

By decentralising passive surveillance across different levels of the health care system, HAT elimination programmes across Africa have undergone an unprecedented transformation. The new strategy enables access to serological screening in frontline rural health facilities but limitations of the screening test requires patients to then undertake onwards referral steps by their own means until they can either be confirmed or discounted as cases. Patients additionally must manage the uncertainty of ancillary health service charges associated with lab testing and hospitalisation at higher facilities. As with many other diseases, diagnosing HAT does not end with the initial test result, but continues throughout treatment pathways with follow-up tests and continual monitoring [54]. Reaching diagnostic consensus is thus the combined result of patient priorities, past precedents, interactions with healthcare providers, and the location and social proximity of services. A HAT surveillance strategy which relies on referral through all levels of the health system will inevitably be subject to the limitations of this system in all of these domains.

In Uganda, a key health system limitation which helps explain referral non-completion appears to be weak communication about HAT testing between health providers and patients. Poor communication meant that some patients did not know they had been tested for HAT when leaving the facility, while others, who did not understand the need for referrals, blamed health staff at both referring and receiving facilities for interpreting tests incorrectly. Such misunderstandings are likely shaped by existing patient knowledge of malaria and prior HAT diagnostic processes which typically lead to immediate treatment. Instead, for patients who test positive by HAT RDTs, the typical outcome is repeated programmatic requests to follow-up discordant results that may fail to result in access to treatment --which is an understandably unsatisfying experience for patients. It also produces potentially iatrogenic effects on the health system by eroding important aspects of trust in both diagnostic technologies and referral structures. Medical historians have shown that elimination success depends on strong health systems [56, 57] but the elimination-health systems relationship can also work the other way around, whereby inappropriate elimination strategies can potentially harm health systems [56]. While Uganda has achieved a high HAT referral completion proportion in the first years under this strategy, effectively addressing health provider communication about the meaning of HAT test results here [35, 42], and elsewhere, could avoid future mistrust of HAT referrals and providers as programmes mature.

## Additional file

Additional file 1: Multilingual abstracts in the six official working languages of the United Nations. (PDF 394 kb)

## Abbreviations

CATT: Card agglutination test for trypanosomiasis
CTC: Capillary tube centrifugation
DNA: Deoxyribonucleic acid
FIND: Foundation for Innovative New Diagnostics
FM: Fluorescence microscopy
GP: Glandular puncture
HAT: Human African trypanosomiasis
IFAT: Indirect immunofluorescent antibody test
ISSEP: Intensified Sleeping Sickness Elimination Programme
LAMP: Loop-mediated Isothermal amplification
PPV: Positive predictive value
RDT: Rapid diagnostic tests
RDT+ MS: Rapid diagnostic test positive microscopy negative
RNA: Ribonucleic acid
TL: Immune trypanolysis test

## Footnotes

1 All HAT medicines are currently donated by the pharmaceutical companies who manufacture them, however, available treatments are complex to administer requiring in-patient admission with additional costs to the health system [5]

2 An exception in some programmes related to a small group of patients in whom follow-up lab teams could not find parasites but seropositive reactions were maintained after their blood was re-tested with the CATT test at serial dilutions to rule out non-specific interference. Such patients required ‘active follow-up’ which could include asking them to present to a referral centre or to a mobile screening team at a later date, but was considered costly and not always done [8].

3 In 2014 the number of facilities receiving RDTs was reduced to 125 based on the distribution of identified cases but increased to 149 in 2015 to include more private clinics and facilities close to refugee camps and the South Sudanese border.

4 LAMP is more sensitive than microscopy and more specific than RDTs but needs to be performed by technicians with a very high level of training in well-equipped facilities. Because these sophisticated molecular diagnostics are yet to be ‘field applicable’ in the routine diagnosis of sleeping sickness at the primary health-care level, the direct benefit to patients remains limited [59].

5 While the CATT test is more cumbersome to use than an RDT, a key advantage of the CATT test format is the ability for lab staff to retest blood at serial dilutions which enables a substantial proportion of patients seropositive on whole blood to be ruled out for further follow-up. Depending on the diagnostic algorithm followed in a programme, those who lose positivity at a ¼ dilution might be ruled out immediately as a non-case whereas those who maintain seropositivity at very low dilutions (1/16) might be managed as if they were cases. This reduces the pool of patients needing long-term follow-up to only those persistently seropositive at mid-level dilutions (such as 1/8) [13, 8, 60, 61]. The relative performance of different RDTs compared to CATT on whole blood or dilutions in a passive screening setting is a matter of ongoing debate [62, 63].
