# Supplementary material for: Integrating innovations: a qualitative analysis of referral non-completion among rapid diagnostic test-positive patients in Uganda’s human African trypanosomiasis elimination programme
Source: Infect Dis Poverty. 2018 Aug 18;7:84. doi: 10.1186/s40249-018-0472-x (PMC6098655; doi:10.1186/s40249-018-0472-x)
Supplement: Supplementary file 1 — Multilingual abstracts in the six official working languages of the United Nations. (PDF 394 kb) [file 40249_2018_472_MOESM1_ESM.pdf]

Translation of the abstract into the six official working languages of the United Nations

تضمين الابتكارات: تحليل نوعي لعدم إكمال الإحالة بين المرضي ذوي النتائج الإيجابية في الفحص التشخيصي السريع في البرنامج الإنساني للقضاء على داء المثقبيات الأفريقي في أوغندا

شونا جاين لي و جينيفر جاي بالمر

الملخص

الخلفية: تمكّن التطورات الأخيرة للفحص التشخيصي السريع(RDTs) لداء المثقبيات الأفريقي (HAT) برنامج القضاء على المرض من تحقيق الامركزية في خدمات الفحص المصلي في المرافق الصحية الميدانية. ومع ذلك ، لا يزال يتعين على المرضى إجراء عدة تدابير للإحالة لإثبات أنفسهم أو لاستبعادهم كحالات. ولما كان الرصد الدقيق للحالات لا يعتمد على أداء التقنيات التشخيصية فحسب، بل على هياكل دعم الإحالة وقرارات المرضى أيضاً، فقد تناولت هذه الدراسة السبب الكامن وراء إخفاق بعض المرضى المشتبه بكونهم موجبي النتائج في الفحص التشخيصي السريع في إكمال عملية الإحالة التشخيصية في غرب النيل في أوغندا.

المنهجية: في الفترة ما بين أغسطس 2013 ويونيو 2015 ، تم فحص 295 من أصل 346 (85٪) مريضاً موجبي النتائج في الفحص التشخيصي السريع بالفحص المجهري مرة واحدة على أقل تقدير؛ تم اكتشاف 10 حالات من بينهم. أجرينا مقابلات مع 20 مريضاً يشتبه بأنهم موجبي النتائج في الفحص التشخيصي السريع لم يكملوا الإحالة (16 منهم لم يحضروا للفحص المجهري الأول ، و 4 لم يعودوا لإجراء الفحص المجهري الثاني) بهدف استبعادهم كحالات بعد حصولهم على نتيجة متناقضة [الفحص التشخيصي السريع إيجابي، ولكن المجهري سلبي النتائج]. تم تحليل المقابلات موضوعياً لدراسة تجارب المرضى في كل خطوة من خطوات عملية الإحالة.

النتائج: ساعدت الاتصالات الضعيفة لمزود الخدمة حول نتائج الفحص التشخيصي السريع لداء المثقبيات الأفريقي في توضيح سبب عدم إكمال الإحالات في عينة هذه الدراسة. إذ أن أغلب المرضى لم يكونوا على علم بأنهم قد خضعوا لاختبار داء المثقبيات الأفريقي حتى تلقوا النتائج، وبعضهم لم يكن يعلم أن نتائج فحصه قد جاءت إيجابية. اختبار داء المثقبيات الأفريقي وعلاجه مجانيان، إلا أن تكاليف الانتقالات ورسوم الخدمات الصحية الإضافية المتوقعة قد جعلت الكثيرين يعدلون عن رأيهم. إلى جانب أن معظم المرضى قد توقعوا أن حصولهم على نتائج إيجابية في الفحص سوف يعني أن عليهم الخضوع للعلاج. كما أن فشل نتائج الفحص التشخيصي السريع في إعطاء تشخيص قاطع دون الحاجة إلى إجراء المزيد من الاختبارات قد دفع البعض إلى التشكيك في خبرة العاملين الصحيين. بالنسبة للأفراد الأربعة الذين فاتتهم الفحص الثاني، أصبح الامتثال لطلبات الإحالة المتكررة أقل جاذبية لهم عندما لم تُقدّم لهم أي نصيحة تشخيصية بديلة أو علاج.

الاستنتاجات: إن إستراتيجية الرصد القائمة على الفحص التشخيصي السريع، والتي تعتمد على الإحالة عبر النظام الصح بجميع مستوياته، تخضع حتمًا للقيود المفروضة عليها. وفي أوغندا ، كانت نقطة الضعف الهيكلية الرئيسية هي سوء قدرة مقدمي الخدمة على توصيل المعلومات للمرضى حول احتمالية تناقض نتائج اختبار داء المثقبيات الأفريقي، وهي النتيجة الأكثر شيوعًا لدى مرضى الفحص المصلي المشتبه بأنهم موجبي النتائج في الفحص التشخيصي السريع ضمن برنامج القضاء على داء المثقبيات الأفريقي. كما أن سوء فهم المريض لمبررات الإحالة ينطوي على مخاطر الإضرار بالثقة في النظام بأكمله، لذا يجب التصدي إلى ذلك في برامج القضاء على المرض.

Translated from English version into Arabic by Eman Shahen, proofread by Heba Kandel, through

TRANSLATORS
WITHOUT BORDERS

整合创新: 乌干达人类非洲锥虫病消除项目中快速诊断阳性且未完成转诊患者的定性分析

Shona Jane Lee, Jennifer J Palmer

摘要
引言: 人类非洲锥虫病（HAT）快速诊断测试（RDTs）的最新发展使消除项目中血清学筛查服务分散到基层卫生机构。然而，患者仍需通过多个转诊步骤方可确诊或排除。因此，准确的监测不仅依赖于诊断技术的性能，而且依赖于转诊支持机构和患者决策。本研究探讨了乌干达西尼罗河地区一些 RDT 阳性疑似患者未完成转诊过程的原因。
方法: 2013 年 8 月至 2015 年 6 月，346 名受试者中 295 名血清快速筛查阳性患者中至少接受过 1 次显微镜镜检，其中包括 10 例确诊病例。我们采访了 20 名未完成转诊的 RDT 阳性疑似患者[16 例未曾接受过镜检，4 例因收到不同结果还未进行第 2 次镜检者（RDT 阳性，镜检阴性）]。通过主题分析访谈以检查每一步转诊的经验。
结果: 非洲锥虫病 RDT 结果提供者与受试者之间沟通不良有助于解释受试者为何未能完成转诊。多数患者直到收到检测结果才知道他们接受了 HAT 检查，部分患者不知道其筛查结果为阳性。尽管 HAT 检测和治疗是免费的，但预期的交通费和辅助医疗服务费使很多人望而却步，大多数患者期望快速检测阳性后就接受治疗。没有进一步的检测，RDT 结果不能作为确诊的依据导致一些人质疑卫生工作者的专业知识。对于错过第 2 次检查的 4 人而言，在没有给出可选择的诊断建议或治疗的情况下，很难让患者接受重复转诊的要求。
结论: 依赖于通过各级卫生系统转诊的基于 RDT 的监测策略不可避免地受到其局限性的影响。在乌干达，一个关键的结构性弱点是关于非洲锥虫病检测结果不一致（这是非洲锥虫病消除项目 RDT 快速检测很常见的检测结果），卫生工作者与患者缺乏合理沟通与说明。患者对转诊理由的误解进而危害到对整个系统的信任度，应在消除项目中予以解决。

Translated from English version into Chinese by Chao Lv, edited by Pin Yang

CHINESE CENTER FOR DISEASE CONTROL AND PREVENTION
NATIONAL INSTITUTE OF PARASITIC DISEASES

Intégrer l'innovation : analyse qualitative des échecs du processus d'aiguillage après diagnostic rapide positif de patients du programme d'éradication de la trypanosomiase humaine africaine en Ouganda

Shona Jane Lee, Jennifer J. Palmer

Résumé
Contexte: Le développement récent des tests de diagnostic rapide (TDR) de la trypanosomiase humaine africaine (THA) permet aux programmes d'éradication de décentraliser les services d'évaluation sérologique vers les établissements de santé de première ligne. Cependant, il ne dispense pas les patients de se conformer aux nombreuses étapes préalables visant à déceler les cas admissibles. Une surveillance adéquate dépend non seulement de la performance des technologies de diagnostic, mais aussi des structures de soutien encadrant l'aiguillage et de la décision des patients. La présente étude se penche sur les raisons pour lesquelles des personnes qui ont reçu un diagnostic positif aux TDR n'ont pas complété le processus d'aiguillage dans la région du Nil occidental, en Ouganda.
Méthode: Entre août 2013 et juin 2015, 295 des 346 personnes (85 %) dont le TDR s'est avéré positif ont subi au moins un examen microscopique ; on a détecté dix cas de THA. Nous avons interviewé 20 personnes qui n'ont pas poursuivi le processus malgré un diagnostic positif au TDR (16 personnes ne se sont pas présentées au premier examen microscopique et quatre ont manqué au deuxième examen visant à les écarter du processus après de premiers résultats discordants (c'est-à-dire avec un TDR positif, mais un premier test microscopique négatif). Une analyse thématique des entrevues a permis d'étudier les expériences vécues à chaque étape du processus d'aiguillage.

Résultats: Dans notre échantillon, les échecs du processus d'aiguillage s'expliquent par une mauvaise communication des résultats des TDR de la THA. La plupart des patients ont appris qu'ils avaient été testés pour la THA en recevant leurs résultats, et certains ignoraient qu'ils avaient reçu un diagnostic positif. Malgré la gratuité du dépistage et du traitement de la THA, les frais de transport et les coûts accessoires liés aux soins de santé en ont découragé plusieurs. La plupart des patients s'attendaient à ce qu'un résultat positif au TDR les mène automatiquement à un traitement. Certains patients dont les résultats aux TDR se sont avérés insuffisants pour établir un diagnostic définitif sans test microscopique complémentaire ont remis en question la compétence des professionnels de la santé. Les quatre personnes qui ont manqué à leur deuxième examen ont estimé que les nombreuses étapes du processus d'aiguillag sont moins attrayantes si on ne leur offre pas de conseils ou de traitements adaptés à leur diagnostic.

Conclusion: Une stratégie de surveillance des TDR basée sur un processus d'aiguillage à tous les niveaux du système de santé se confronte inévitablement à ses propres limites. En Ouganda, la communication lacunaire concernant les résultats discordants dans le dépistage de la THA dans le cadre d'un programme d'éradication, scénario le plus fréquent chez les cas soupçonnés d'être atteints de la maladie, constitue une faiblesse structurelle de taille. Il faut absolument veiller à ce que les patients comprennent toutes les étapes du processus d'aiguillage pour éviter de miner la confiance qu'ils ont envers le système de santé au grand complet.

Translated from English version into French by Patricia Barthélémy, proofread by Sophie N, through

TRANSLATORS
WITHOUT BORDERS

Внедрение инноваций: качественный анализ случаев незавершённого обследования среди пациентов с положительным результатом экспресс-теста в рамках программы по борьбе с африканским трипаносомозом человека в Уганде.

Шона Джейн Ли, Дженнифер Джей Палмер

Аннотация

Исходная информация: Новейшие разработки в области экспресс-тестирования (ЭТ) на африканский трипаносомоз человека (АТЧ) в рамках элиминационных программ позволяют передавать серологические исследования полевым медицинским пунктам. Несмотря на это, пациенты, после получения рекомендаций по обследованию должны предпринимать дополнительные действия для подтверждения или опровержения диагноза. Таким образом, эффективность наблюдения определяется не только технологиями диагностики, но и последующим медицинским обслуживанием и решениями, принимаемыми пациентом. Цель настоящего исследования – выяснить, почему некоторые пациенты с положительным результатом ЭТ не смогли завершить обследование после получения медицинского направления в провинции Западный Нил, Уганда.

Методы: В период с августа 2013 г. по июнь 2015 г. 295/346 (85%) людей с положительным результатом ЭТ прошли обследование методом микроскопии, по крайней мере, один раз; выявлено 10 случаев заболевания. Мы опросили 20 пациентов с положительным результатом ЭТ, не завершивших процедуру диагностики (16 не прошли первую микроскопию, а 4 не обратились повторно для уточнения спорных результатов [положительный результат

ЭТ, отрицательный результат микроскопии]). Для изучения особенностей каждого этапа обследования данные анализировались тематически.
Результаты: Плохая осведомленность о положительном результате ЭТ на АТЧ по вине медицинских работников в указанных случаях. Большинство пациентов не были проинформированы о том, что они подвергались тестированию на АТЧ до тех пор, пока не пришли результаты, а некоторых не известили об их положительном результате. Хотя диагностика и лечение АТЧ бесплатны, многих отпугивали предполагаемые расходы на дорогу и на дополнительные медицинские услуги. Большинство пациентов считали, что положительный результат ЭТ предполагает лечение от АТЧ. Некоторые пациенты усомнились в компетентности медицинских работников, поскольку для постановки точного диагноза кроме результатов ЭТ, понадобились дополнительные мероприятия. Четыре пациента, которые не явились на второе обследование, не согласились проходить дообследование, так как дополнительной диагностики и лечения не было предложено.
Выводы: Стратегия надзора посредством ЭТ, основанная на проведении обследований на всех уровнях системы здравоохранения, неизменно подвергается ограничениям. В Уганде основная структурная недоработка заключалась в недостаточной осведомлённости пациентов по вине медицинских работников о возможных противоречивых результатах теста на АТЧ, что является наиболее частым исходом серологических исследований методом ЭТ в рамках программы по борбе с АТЧ. Недостаточная осведомлённость пациентов о целесообразности завершения обследования может привести к подрыву доверия к системе здравоохранения в целом и должна быть учтена в элиминационных программах.

Translated from English version into Russian by Dream Translator, proofread by Tatiana Kary, through

Innovaciones integradoras: un análisis cualitativo de las derivaciones no finalizadas entre pacientes diagnosticados positivos mediante prueba rápida en el programa de erradicación de la tripanosomiasis africana humana en Uganda.

Shona Jane Lee, Jennifer J. Palmer

Resumen
Contexto: el reciente desarrollo de las pruebas de diagnóstico rápido (PDR) para la tripanosomiasis africana humana (TAH) posibilita a los programas de erradicación la descentralización de los servicios de cribado serológico de las instalaciones sanitarias de la primera línea. Sin embargo, los pacientes aún deben comprometerse a seguir los múltiples pasos de derivación para ser o confirmados o descartados como casos. Por consiguiente, la exactitud de la vigilancia no depende solo del desempeño de las tecnologías de diagnóstico, sino también de las estructuras de apoyo de la derivación y de las decisiones del paciente. Este estudio ha analizado por qué algunos sujetos con PDR positivas no completaron el proceso de diagnóstico de derivación en el distrito del Nilo Occidental, Uganda.
Metodología: entre agosto de 2013 y junio de 2015, 295 personas (las cuales dieron positivo en las PDR) de un total de 346 (85 %) fueron examinadas con microscopia al menos una vez. Se detectaron 10 casos. Entrevistamos a 20 sujetos con PDR positivas que no habían completado la derivación (16 de ellas no se habían presentado a la primera exploración con microscopia y 4 no habían regresado para la segunda para ser descartados como casos tras recibir discordancia [PDR

positivas y resultados de microscopia negativos]). Las entrevistas se analizaron en grupos temáticos para examinar experiencias de cada etapa del proceso de derivación.

Resultados: la pobre comunicación de los profesionales sanitarios sobre los resultados de las PDR de la TAH ayudó a explicar las derivaciones no finalizadas en nuestra muestra. La mayoría de los pacientes no eran conscientes de que eran examinados de TAH hasta que recibían los resultados y otros no sabían que habían dado positivo. Aunque la prueba y el tratamiento de la TAH es gratuito, los costes anticipados de transporte y las tasas de los servicios sanitarios auxiliares disuadieron a muchos. La mayor previsión de un resultado positivo de las PDR derivaría en el tratamiento de la TAH. Los resultados de las PDR que fracasaron en proveer un diagnóstico definitivo por falta de pruebas nos llevan a cuestionar la pericia de los trabajadores sanitarios. Para los cuatro sujetos que no fueron a la segunda exploración, cumplir con las reiteradas solicitudes de derivación era menos atractivo cuando no se les daba consejo o tratamiento diagnóstico alternativo.

Conclusión: una estrategia de vigilancia basada en las PDR que depende de la derivación a lo largo de todos los niveles del sistema sanitario está sujeta a limitaciones inevitables. En Uganda, una debilidad estructural clave fue la pobre comunicación proveída acerca de la posibilidad de resultados discordantes de las pruebas de la TAH, lo cual es el resultado más común para los sujetos de PDR serológica en un programa de erradicación de la TAH. La lógica confusión de un paciente derivado perjudica la confianza en el sistema en su totalidad y debería solventarse en los programas de erradicación.

Translated from English version into Spanish by Fran Egea, proofread by Maria Alejandra Aguada, through

TRANSLATORS
WITHOUT BORDERS
